# Peroxisome proliferator-activated receptor γ (PPARγ) suppresses the proliferation and metastasis of patients with urothelial carcinoma after renal transplantation by inhibiting LEF1/β-catenin signaling

**DOI:** 10.1080/21655979.2020.1843834

**Published:** 2020-12-08

**Authors:** Donghao Shang, Yuting Liu, Jian Zhang, Xinyi Hu

**Affiliations:** a Department of Urology, Beijing Friendship Hospital, Capital Medical University, Beijing, China; b Department of Pathology, Capital Medical University, Beijing, China

**Keywords:** PPARγ, LEF1/β-catenin signaling, urothelial carcinoma, renal transplantation

## Abstract

This study is to investigate the role of peroxisome proliferator-activated receptor γ (PPARγ) in the progression of urothelial carcinoma (UC) after renal transplants (RT). A total of 114 UC patients were gathered, including 60 cases of primary UC and 54 cases UC after RT. RT-PCR was used to detect the mRNA expression of the 54 patients with UC after RT, and immunohistochemistry and western blot were used to examine the protein expression. The proliferative ability of two UC cell lines, and 5637, were measured by WST-1 assay. Transwell system was used to analyze the migration and invasion of UC cells. PPARγ agonist Rosiglitazone and the antagonist GW9662 were used to alter the PPARγ expression. siRNA targeting LEF1 and expression vector containing full-length cDNA of LEF1 regulated the expression of LEF1. Pathway analysis indicated that PPARγ expression was significantly down regulated. Compared with normal urothelium and primary UC, the expression of PPARγ in UC was significantly decreased in RT group. PPARγ expression was correlated with tumor size, clinical stage, pathological and recurrence. PPARγ inactivates LEF1/β-catenin signaling in UC cells. PPARγ decreased the protein expression of MMP2, and calpain-2. PPARγ suppresses the proliferation, and invasion of UC cells depending on the expression of LEF1. PPARγ inhibited tumor proliferation and metastasis by inhibiting LEF1/β-catenin signaling, and the expression of PPARγ in UC after RT decreased significantly. Our findings also suggested that PPARγ may be a potential biomarker for the diagnosis of UC after RT.

## Graphical abstract

**Figure:**
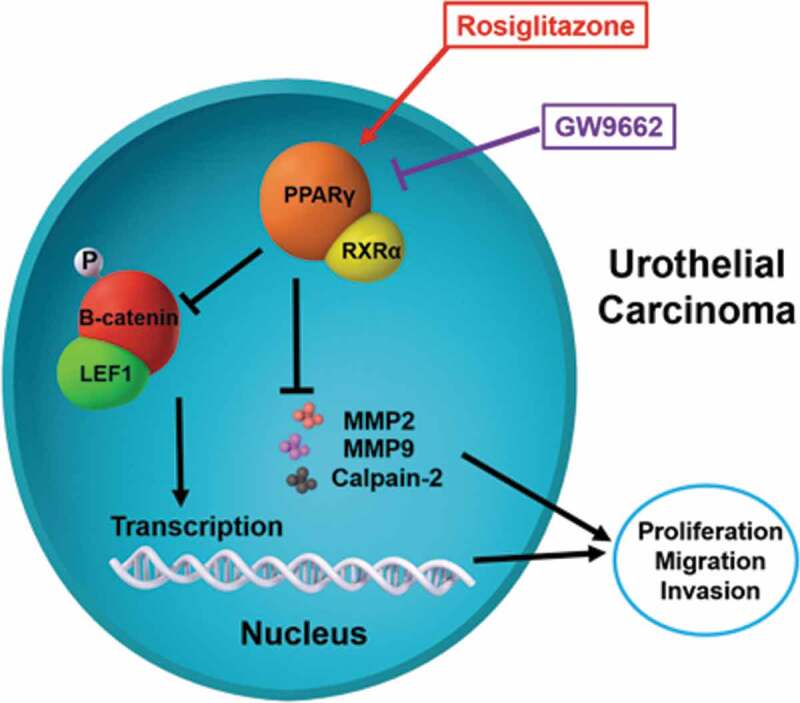

## Introduction

The molecular mechanism of UC after RT remains unclear. Based on our previous finding, compared with normal urothelial tissues, 1597 mRNAs in UC were up-regulated and 1032 mRNAs were down-regulated. PPAR signaling may be involved in UC after RT [1]. PPARs act as nuclear hormone receptors and regulate various biological processes. For example, lipid accumulation, fatty and glucose metabolism, cell and proliferation, deregulation of PPARs. Except for this, PPARs are related to the development of human tumors. Recent studies have shown that PPAR-β/γ is a tumor suppressor can inhibit the growth of breast cancer cells. PPAR-β/γ may also inhibit the proliferation of gland cancer cells by increasing apoptosis. As the most important protein in PPARs, the details of PPARγ functions in UC progression are still indistinct. In addition, in many living mammalian cells, PPARγ, and the typical Wnt/beta-catenin pathway exhibit opposite behavior.

LEF1 belongs to a member of the lymphoid-enhancing factor/T-cell factor (LEF/TCF) family [2]. As a transcription factors, it regulates gene expression and coordinates cellular processes through the Wnt/β-catenin signaling pathway [3,4]. Matrix metalloproteinases (MMPs) belong to the family of zinc-dependent endopeptidases and can be divided into several types [5]. MMP overexpressed in various human malignancies can degrade matrix as their name suggests and maintain the homeostatic regulation of the extracellular environment [6]. MMP2 and MMP9 exist in gelatinases and shown to be related cell migration and invasion [6,7]. Calpain-2 is a thiol proteinase, consisting of a catalytic subunit and a regulatory subunit [8]. Calpain-2 is activated by Ca^2+^ at the millimolar level and has been reported to mediate the invasion of glioma cells [9]. However, the effect of PPARγ on calpain-2, MMP2 and MMP9 in urothelial carcinoma is unknown and remains to be elucidated.

**Figure 1.**
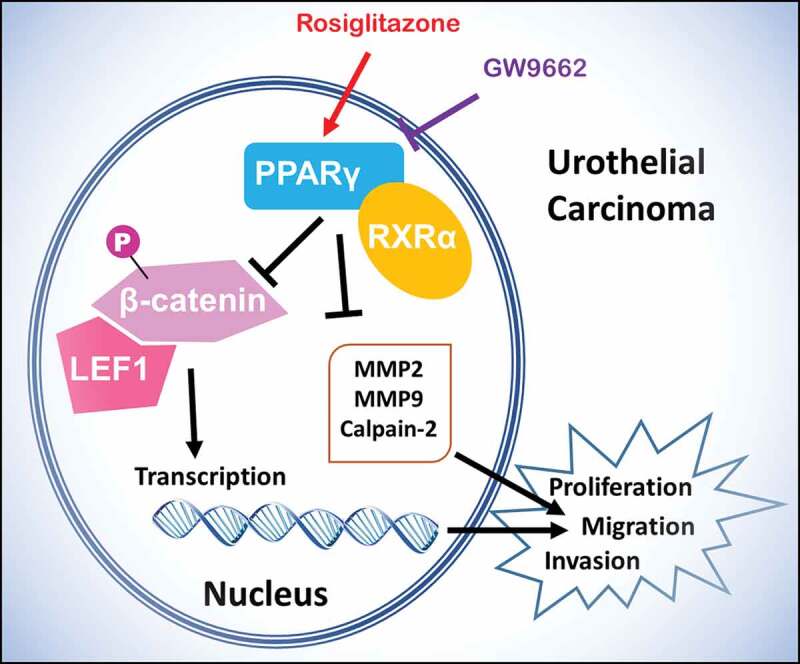
Scheme of PPARγ function and regulation of LEF1/phosphor-β-catenin and MMP2, MMP9 and calpain-2 in urothelial carcinoma. Rosiglitazone is PPARγ agonist, and GW9662 is PPARγ antagonist

In this study, the PPARγ agonist Rosiglitazone and the antagonist GW9662 was used to alter the PPARγ expression (Figure 1). The expression level of mentioned proteins, as well as the proliferation, and invasion of treated UC cells were detected.

## Materials and methods

### Tumor specimens

A total of 114 tumor specimens from patients with UC admitted to Department of Urology, Beijing Friendship Hospital from 2005 to 2018 were collected. Among them, 54 cases were diagnosed as UC after RT and 60 patients were primary UC. The histological cell types of all tumor specimens were evaluated by two experienced pathologists. Before specimen collection, all the patients with UC did not receive any treatment. In order to evaluate the expression of PPARγ primary UC cases were selected and equivalent to UC after RT in the stage and pathological grade. Specimens preserved in 10% formalin were embedded in paraffin for immunohistochemistry staining, and serially sectioned onto microscope slides at a thickness of 4 μm. Keeping other specimens in liquid nitrogen to extract mRNA.

### Ethics statement

The study was approved by Ethics Boards of Beijing Friendship Hospital, Capital Medical University. All tissue sample acquisition was carried out according to the institutional guidelines. All the patients approved to take part in our study and issued written informed consents.

### Cell cultures and agents

The American Type Culture Collection (Rockville, MD, USA) provides two UC cell lines T24 and 5637. UC cells were cultured in RPMI 1640 medium (Gibco, Bio-Cult Diagnostics Limited, Glasgow, UK) supplemented with 10% FBS (100 U/ and streptomycin, 100 units/ml penicillin, 100 mg/ and 1% nonessential amino acids). All UC cell lines were incubated in a humidified atmosphere containing 5% CO_2_ at 37℃. The PPARγ agonist Rosiglitazone, and the antagonist GW9662 were purchased from Selleck Chemicals (TX, USA).

### Kyoto encyclopedia of genes and genomes (KEGG) pathway analysis

**Figure 2.**
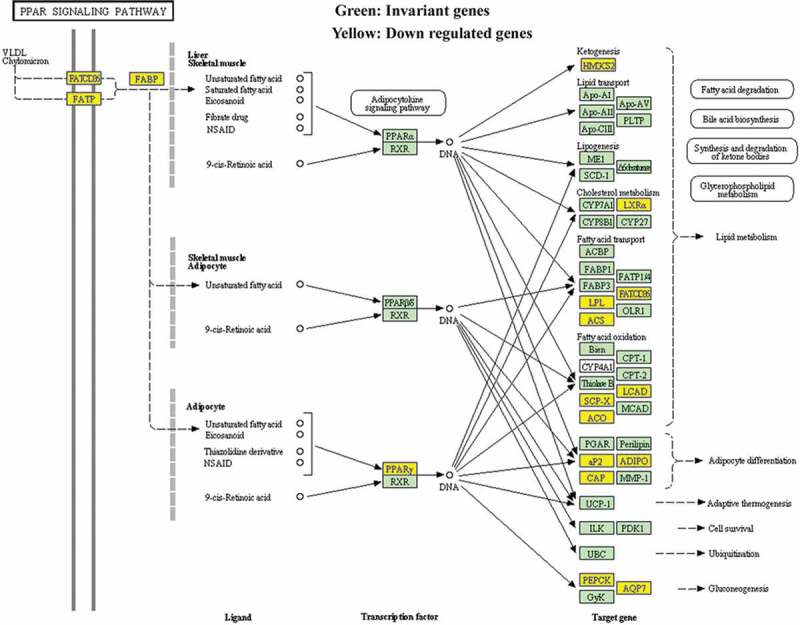
The pathway map of PPAR signaling in patients with UC after RT was selected using the KEGG pathway analysis

**Figure 3.**
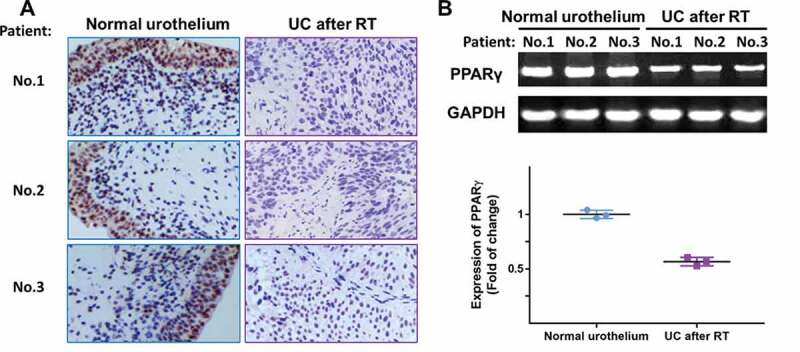
The expression of PPARγ was examined in specimens of normal urothelium and UC after RT using immunohistochemistry (a) and RT-PCR (b). Three representative images were shown

Volcano plot filtered the mRNA of RT recipients in UC and corresponding RT recipients in normal (Figure 2), and using the KEGG pathway analysis in Figure 3 and Table 1 to further study the key pathways associated with carcinogenesis of UC after RT.

**Table 1.** The key pathway used for the KEGG enrichment analyses

PathwayID	Definition	OriginalWebSite	Fisher-Pvalue	SelectionCounts	SelectionSize	Count	Size	FDR	Enrichment_Score	Genes
hsa03320	PPAR signaling pathway – Homo sapiens (human)	http://www.genome.jp/kegg-bin/show_pathway?hsa03320+33+8309+2180+9370+364+948+2167+3158+4023+10062+5105+5468+6342+11001+10580	1.26032E-06	15	342	69	6756	0.00035415	5.899519	ACADL//ACOX2//ACSL1//ADIPOQ//AQP7//CD36//FABP4//HMGCS2//LPL//NR1H3//PCK1//PPARG//SCP2//SLC27A2//SORBS1
hsa00071	Fatty acid degradation – Homo sapiens (human)	http://www.genome.jp/kegg-bin/show_pathway?hsa00071+10449+33+2180+124+125+126+130+217+223+10455+3033	8.09338E-06	11	342	44	6756	0.00113712	5.09187	ACAA2//ACADL//ACSL1//ADH1A//ADH1B//ADH1C//ADH6//ALDH2//ALDH9A1//ECI2//HADH
hsa00982	Drug metabolism – cytochrome P450 – Homo sapiens (human)	http://www.genome.jp/kegg-bin/show_pathway?hsa00982+124+125+126+130+316+1576+1577+2327+2330+4128+4129+4257	0.000130432	12	342	68	6756	0.01221709	3.884617	ADH1A//ADH1B//ADH1C//ADH6//AOX1//CYP3A4//CYP3A5//FMO2//FMO5//MAOA//MAOB//MGST1
hsa00561	Glycerolipid metabolism – Homo sapiens (human)	http://www.genome.jp/kegg-bin/show_pathway?hsa00561+84803+217+223+84649+1607+1608+57678+4023+11343+8613	0.000368266	10	342	55	6756	0.02587068	3.433838	AGPAT9//ALDH2//ALDH9A1//DGAT2//DGKB//DGKG//GPAM//LPL//MGLL//PPAP2B
hsa00350	Tyrosine metabolism – Homo sapiens (human)	http://www.genome.jp/kegg-bin/show_pathway?hsa00350+124+125+126+130+316+1638+4128+4129	0.000614481	8	342	39	6756	0.02890198	3.211491	ADH1A//ADH1B//ADH1C//ADH6//AOX1//DCT//MAOA//MAOB
hsa04610	Complement and coagulation cascades – Homo sapiens (human)	http://www.genome.jp/kegg-bin/show_pathway?hsa04610+716+718+725+730+4179+1675+2159+2160+2162+2157+7035	0.000617124	11	342	69	6756	0.02890198	3.209627	C1S//C3//C4BPB//C7//CD46//CFD//F10//F11//F13A1//F8//TFPI
hsa00830	Retinol metabolism – Homo sapiens (human)	http://www.genome.jp/kegg-bin/show_pathway?hsa00830+124+125+126+130+216+316+1562+1576+1577+54884	0.001271915	10	342	64	6756	0.05105831	2.895542	ADH1A//ADH1B//ADH1C//ADH6//ALDH1A1//AOX1//CYP2C18//CYP3A4//CYP3A5//RETSAT
hsa00010	Glycolysis/Gluconeogenesis – Homo sapiens (human)	http://www.genome.jp/kegg-bin/show_pathway?hsa00010+124+125+126+130+217+223+5105+5160+5224+5232	0.001820027	10	342	67	6756	0.06392844	2.739922	ADH1A//ADH1B//ADH1C//ADH6//ALDH2//ALDH9A1//PCK1//PDHA1//PGAM2//PGK2
hsa00260	Glycine, serine and threonine metabolism – Homo sapiens (human)	http://www.genome.jp/kegg-bin/show_pathway?hsa00260+1491+29958+2628+4128+4129+5224+6470	0.00259477	7	342	38	6756	0.08101449	2.585901	CTH//DMGDH//GATM//MAOA//MAOB//PGAM2//SHMT1
hsa05144	Malaria – Homo sapiens (human)	http://www.genome.jp/kegg-bin/show_pathway?hsa05144+948+2995+3039+3040+3043+22914+7058+7412	0.002897191	8	342	49	6756	0.08141108	2.538023	CD36//GYPC//HBA1//HBA2//HBB//KLRK1//THBS2//VCAM1
hsa00380	Tryptophan metabolism – Homo sapiens (human)	http://www.genome.jp/kegg-bin/show_pathway?hsa00380+217+223+316+847+3033+4128+4129	0.003514375	7	342	40	6756	0.08977632	2.454152	ALDH2//ALDH9A1//AOX1//CAT//HADH//MAOA//MAOB
hsa00020	Citrate cycle (TCA cycle) – Homo sapiens (human)	http://www.genome.jp/kegg-bin/show_pathway?hsa00020+47+48+3417+5105+5160+8801	0.004040926	6	342	31	6756	0.09462501	2.393519	ACLY//ACO1//IDH1//PCK1//PDHA1//SUCLG2
hsa00280	Valine, leucine and isoleucine degradation – Homo sapiens (human)	http://www.genome.jp/kegg-bin/show_pathway?hsa00280+10449+217+223+316+3033+3158+5095	0.00607465	7	342	44	6756	0.1313059	2.216479	ACAA2//ALDH2//ALDH9A1//AOX1//HADH//HMGCS2//PCCA
hsa05204	Chemical carcinogenesis – Homo sapiens (human)	http://www.genome.jp/kegg-bin/show_pathway?hsa05204+124+125+126+130+1562+1576+64816+1577+3290+4257	0.006756753	10	342	80	6756	0.1356177	2.170262	ADH1A//ADH1B//ADH1C//ADH6//CYP2C18//CYP3A4//CYP3A43//CYP3A5//HSD11B1//MGST1
hsa04146	Peroxisome – Homo sapiens (human)	http://www.genome.jp/kegg-bin/show_pathway?hsa04146+225+8309+2180+847+10455+3295+3417+55825+6342+11001	0.007372504	10	342	81	6756	0.1381116	2.132385	ABCD2//ACOX2//ACSL1//CAT//ECI2//HSD17B4//IDH1//PECR//SCP2//SLC27A2
hsa00650	Butanoate metabolism – Homo sapiens (human)	http://www.genome.jp/kegg-bin/show_pathway?hsa00650+348158+6296+54988+3033+3158	0.008807746	5	342	26	6756	0.1422708	2.055135	ACSM2B//ACSM3//ACSM5//HADH//HMGCS2
hsa04614	Renin-angiotensin system – Homo sapiens (human)	http://www.genome.jp/kegg-bin/show_pathway?hsa04614+185+1215+2028+4311	0.009084056	4	342	17	6756	0.1422708	2.04172	AGTR1//CMA1//ENPEP//MME
hsa01230	Biosynthesis of amino acids – Homo sapiens (human)	http://www.genome.jp/kegg-bin/show_pathway?hsa01230+48+445+1491+84706+3417+5009+5224+5232+6470	0.00911,3432	9	342	71	6756	0.1422708	2.040318	ACO1//ASS1//CTH//GPT2//IDH1//OTC//PGAM2//PGK2//SHMT1
hsa01212	Fatty acid metabolism – Homo sapiens (human)	http://www.genome.jp/kegg-bin/show_pathway?hsa01212+10449+33+2180+60481+3033+55301+55825	0.009821419	7	342	48	6756	0.1452536	2.007826	ACAA2//ACADL//ACSL1//ELOVL5//HADH//OLAH//PECR
hsa00360	Phenylalanine metabolism – Homo sapiens (human)	http://www.genome.jp/kegg-bin/show_pathway?hsa00360+10249+4128+4129+9588	0.0112222	4	342	18	6756	0.1547451	1.949922	GLYAT//MAOA//MAOB//PRDX6
hsa00980	Metabolism of xenobiotics by cytochrome P450 – Homo sapiens (human)	http://www.genome.jp/kegg-bin/show_pathway?hsa00980+124+125+126+130+874+1576+1577+3290+4257	0.01184989	9	342	74	6756	0.1547451	1.926286	ADH1A//ADH1B//ADH1C//ADH6//CBR3//CYP3A4//CYP3A5//HSD11B1//MGST1
hsa00340	Histidine metabolism – Homo sapiens (human)	http://www.genome.jp/kegg-bin/show_pathway?hsa00340+217+223+3176+4128+4129	0.01211527	5	342	28	6756	0.1547451	1.916667	ALDH2//ALDH9A1//HNMT//MAOA//MAOB
hsa04062	Chemokine signaling pathway – Homo sapiens (human)	http://www.genome.jp/kegg-bin/show_pathway?hsa04062+6346+6358+6359+10344+6348+6349+414062+6351+388372+6355+6387+2920+9844+2869+56288+5196+5197	0.01483876	17	342	189	6756	0.1812909	1.828602	CCL1//CCL14//CCL15//CCL26//CCL3//CCL3L1//CCL3L3//CCL4//CCL4L1//CCL8//CXCL12//CXCL2//ELMO1//GRK5//PARD3//PF4//PF4V1
hsa00564	Glycerophospholipid metabolism – Homo sapiens (human)	http://www.genome.jp/kegg-bin/show_pathway?hsa00564+84803+1607+1608+57678+2819+23171+162466+11145+5320+8613	0.01617805	10	342	91	6756	0.189418	1.791074	AGPAT9//DGKB//DGKG//GPAM//GPD1//GPD1L//PHOSPHO1//PLA2G16//PLA2G2A//PPAP2B
hsa04620	Toll-like receptor signaling pathway – Homo sapiens (human)	http://www.genome.jp/kegg-bin/show_pathway?hsa04620+6348+6349+414062+6351+388372+2353+3446+3449+3441+7098+7100	0.01782412	11	342	106	6756	0.2003431	1.748992	CCL3//CCL3L1//CCL3L3//CCL4//CCL4L1//FOS//IFNA10//IFNA16//IFNA4//TLR3//TLR5
hsa00330	Arginine and proline metabolism – Homo sapiens (human)	http://www.genome.jp/kegg-bin/show_pathway?hsa00330+217+223+445+2628+4128+4129+5009	0.02612399	7	342	58	6756	0.2823401	1.58296	ALDH2//ALDH9A1//ASS1//GATM//MAOA//MAOB//OTC
hsa00630	Glyoxylate and dicarboxylate metabolism – Homo sapiens (human)	http://www.genome.jp/kegg-bin/show_pathway?hsa00630+48+847+5095+6470	0.03071557	4	342	24	6756	0.3134913	1.512641	ACO1//CAT//PCCA//SHMT1
hsa04974	Protein digestion and absorption – Homo sapiens (human)	http://www.genome.jp/kegg-bin/show_pathway?hsa04974+7373+255631+1290+1357+1803+4311+643834+643847+5222	0.03317146	9	342	88	6756	0.3134913	1.479235	COL14A1//COL24A1//COL5A2//CPA1//DPP4//MME//PGA3//PGA4//PGA5
hsa04060	Cytokine-cytokine receptor interaction – Homo sapiens (human)	http://www.genome.jp/kegg-bin/show_pathway?hsa04060+6346+6358+6359+10344+6348+6349+414062+6351+388372+6355+6387+2920+2277+3446+3449+3441+3953+3977+80310+5196+5197	0.03328975	21	342	271	6756	0.3134913	1.477689	CCL1//CCL14//CCL15//CCL26//CCL3//CCL3L1//CCL3L3//CCL4//CCL4L1//CCL8//CXCL12//CXCL2//FIGF//IFNA10//IFNA16//IFNA4//LEPR//LIFR//PDGFD//PF4//PF4V1
hsa00750	Vitamin B6 metabolism – Homo sapiens (human)	http://www.genome.jp/kegg-bin/show_pathway?hsa00750+316+493911	0.03346882	2	342	6	6756	0.3134913	1.47536	AOX1//PHOSPHO2
hsa04080	Neuroactive ligand-receptor interaction – Homo sapiens (human)	http://www.genome.jp/kegg-bin/show_pathway?hsa04080+150+153+154+185+885+1131+51083+2897+3062+3356+3953+4828+4886+4889+2908+4922+4988+9934+56288+5733+5737+1901+6863+6866	0.03459132	24	342	321	6756	0.3135536	1.461033	ADRA2A//ADRB1//ADRB2//AGTR1//CCK//CHRM3//GAL//GRIK1//HCRTR2//HTR2A//LEPR//NMB//NPY1R//NPY5R//NR3C1//NTS//OPRM1//P2RY14//PARD3//PTGER3//PTGFR//S1PR1//TAC1//TAC3
hsa04623	Cytosolic DNA-sensing pathway – Homo sapiens (human)	http://www.genome.jp/kegg-bin/show_pathway?hsa04623+6351+388372+3446+3449+3441+10622+84265	0.04181909	7	342	64	6756	0.3672239	1.378625	CCL4//CCL4L1//IFNA10//IFNA16//IFNA4//POLR3G//POLR3GL

### Immunohistochemistry

The slides were deparaffinized in xylene and then rehydrated in a graded series of ethanol, followed by antigen retrieval in a microwave. To block the activity of endogenous peroxidase, the slides were incubated with 10% rabbit serum and 3% hydrogen peroxide for 10 min at room temperature. Then, the slides were incubated with a PPAR gamma antibody (ab59256, Abcam, Cambridge, UK; 1:500) overnight at 4℃. Then, washing twice in Tris-buffered saline and incubating the slides with goat anti-rabbit polymers (E0432, Dako, Glostrup, Denmark) at rt for. Using a standard streptavidin-biotin complex (Sigma, MO, USA) for the final test and using Olympus BH2 microscope (Olympus, Tokyo, Japan) to evaluate the results.

### RT-PCR and quantitative RT-PCR

Total RNA was extracted from frozen surgical specimens and UC cell lines using Trizol reagent (Invitrogen, Carlsbad, USA). it was reverse transcripted into cDNA by random primers and M-MLV reverse transcriptase (Tiangen, Beijing, China). RNA quality was evaluated by electrophoresis on a 1% agarose gel. PCR Master Mix kit (Tiangen, Beijing, China) was used for cDNA amplification, and quantitative real-time PCR was performed by a Mastercycler real-time PCR machine (Eppendorf, Hamburg, Germany) using SYBR Green kit (Applied Biosystems, CA, USA) according to the manufacturer’s instruction. The relative mRNA expression levels were analyzed using the 2^–ΔΔCt^ method. All sequences (Table 2) were achieved by Primer Premier 5.0 software and GAPDH acted as an internal control.

**Table 2.** Sequences of primers and siRNA oligonucleotides

Primers	Forward primer (5'-3')	Reverse primer (5'-3')	Length of PCR products (bp)
PPARγ	TAGTCGAGGCACCTAGAGA	CTTGTGAATGGAATGTCTTCG	122
RXRα	TGACGTGCGACGTCGACAA	ACCTTGAGGACGCCATTGAG	110
GAPDH	GAAGGTGAAGGTCGGAGTC	GAAGATGGTGATGGGATTTC	226
*siRNA*	*Sense oligonucleotide (5'-3')*	*Antisense oligonucleotide (5'-3')*	*Target gene sequence (5'-3')*
LEF1	AAGAGAAAGAGAAGUUUGCC	GCAAACUUCUCUUUCUCUUCC	TGGCAAACTTCTTTCTCTTCT
Negative control	GUACCGCACGUCAUUCGUAUC	UACGAAUGACGUGCGGUACGU	

### Western blotting

Treating tissues and UC cells with 500 μL of cell lysis buffer (Cell Signaling, Cambridge, UK), and the protein concentration was measured by ELx800 spectrophotometer (BIO-TEK™; Wolf Laboratories, York, UK) using DC Protein Assay kit (Bio-Rad, Hemel Hemstead, England, UK). 50 ng of protein was separated by sodium dodecyl sulfate-polyacrylamide gel electrophoresis and transferred into PVDF membrane. 2 hours after immunostaining with primary antibody, membrane was incubated with HRP-conjugated secondary antibody for 4 h Finally, protein bands were visualized using the enhanced chemiluminescence (ECL) system (Amersham, Aylesbury, UK), and photographed using an UVITech imager (UVITech, Inc., Cambridge, UK). LEF1 (# C12A5) rabbit mAb, β-catenin (# D10A8) XP® rabbit mAb, phospho-β-catenin (Ser33/37), MMP-2 (# D8N9Y) rabbit mAb, MMP-9 (# D6O3H) XP® rabbit mAb and anti-rabbit HRP-linked antibody (# 7074) were purchased from Cell Signaling Technology. PPAR gamma antibody (# ab59256), calpain 2 antibody (# ab39165) and β-actin monoclonal antibody (# ab6276) were purchased from Abcam (Cambridge, UK).

### Cell transfections

siDirect software was used to design the siRNA oligonucleotides for LEF1 (Table 2). The negative control used a scramble siRNA. UC cells were cultured until the cell confluence reached to 50%. Then, UC cells transfected with LEF1 siRNA (siLEF1) or scramble siRNA (Negative siRNA) respectively. Additionally, UC cells were stably transfected either with the expression vector containing full-length cDNA for LEF1 or a blank vector without LEF1 insert. In the light of manufacturer’s regulations, all transfections were performed with Lipofectamine 2000 (Invitrogen Life Technologies, Carlsbad, CA, USA). Monoclonal colonies were selected by G418, and UC cells were collected to detect the expression of LEF1 by WB.

### Cell proliferation assay

According to the manufacturer’s regulations, the proliferative ability of UC cells was evaluated by WST-1 analysis. After different treatments, the UC cells were plated in the 96-well plate. After incubation for 72 hours, 20 μL of WST-1 (Roche, Penzberg, Germany) was added into each well. After 2 hours, the absorbance was measured by an enzyme-labeled analyzer at 450 nm (Immunoreader, Tokyo, Japan).

### Cell migration and invasion assay

For cell migration assay, polyethylene terephthalate membrane (8 μm-pores) was installed in 24-well transwell plates chamber (Corning, NY, USA). For cell invasion assay, the top chambers were coated with 30 μL of ECM (Extracellular matrix; Sigma, USA). The top chambers were filled with UC cells (2 × 10^5^) in serum-free medium and the bottom chambers were filled with medium (10% FBS). The cells were incubated at 37℃ in 5% CO_2_ for 12 h, then chamber membrane was fixed with 4% paraformaldehyde and stained with crystal violet. 10 high power fields were randomly selected under Olympus BH2 microscope (Olympus, Tokyo, Japan) to count the migration or invasion cells.

### Statistical analysis

All statistical analyses in this study were performed using SPSS version 18.0 software (IBM, USA). Categorical data were analyzed using the chi-square test. Data were analyzed using independent two-tailed t-test, and all experiments were performed in triplicate. Data was presented as mean ± standard deviation. *P* < 0.05 were considered statistically significant.

## Results

### KEGG pathway analysis

Based on the previous profiling and microarray analysis of mRNA associated with UC after RT [1], the major pathways involved in the carcinogenesis were further evaluated by conducting KEGG pathway analysis. The result in Table 3 showed that several signaling pathways were involved in the down regulation of UC associated mRNAs, including PPAR, fatty acid degradation, drug metabolism – cytochrome P450, glycerolipid metabolism, tyrosine metabolism, complement, and coagulation cascades, retinol metabolism, glycolysis/gluconeogenesis, glycine, serine and threonine metabolism, and malaria (P < 0.001 for each pathway). Particularly, PPAR signaling pathway exhibited the highest selection counts and enrichment score.

**Table 3.** Signaling pathways involved in the down regulation of UC associated mRNAs by KEGG pathway analysis

Pathway ID	Definition	Fisher-P value	Selection Counts	Count	Enrichment Score
hsa03320	PPAR signaling pathway	1.26E-06	15	69	5.90
hsa00071	Fatty acid degradation	8.09E-06	11	44	5.09
hsa00982	Drug metabolism – cytochrome P450	1.30E-04	12	68	3.88
hsa00561	Glycerolipid metabolism	3.68E-04	10	55	3.43
hsa00350	Tyrosine metabolism	6.14E-04	8	39	3.21
hsa04610	Complement and coagulation cascades	6.17E-04	11	69	3.21
hsa00830	Retinol metabolism	1.27E-03	10	64	2.90
hsa00010	Glycolysis/Gluconeogenesis	1.82E-03	10	67	2.74
hsa00260	Glycine, serine and threonine metabolism	2.59E-03	7	38	2.59
hsa05144	Malaria	2.90E-03	8	49	2.54

**Figure 4.**
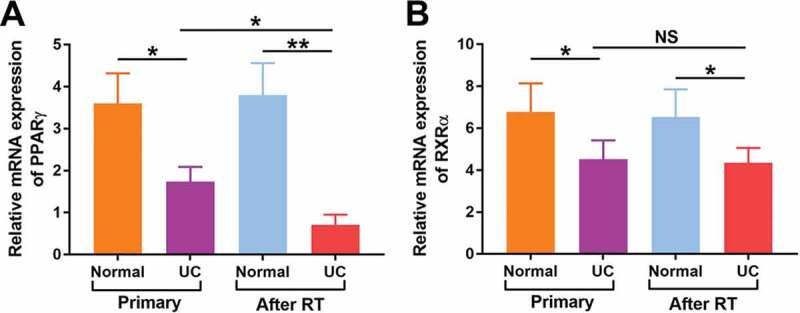
Relative mRNA levels of PPARγ (a) and RXRα (b) were detected in both normal urothelium and UC of patients with primary UC and recipients with UC after RT by quantitative RT-PCR. *p < 0.05; **p < 0.01

Therefore, pathway map of PPAR signaling was analyzed and drawn (Figure 4). Down-regulated genes were marked by yellow nodes and invariant genes without difference were marked by green nodes. Compared to normal urothelial tissues, a significant down regulation of PPARγ further leading to several down-regulated target genes in UC after RT could be observed.

### Patient characteristics

In this study, a total of 114 patients with UC including 60 patients with primary UC and 54 patients with UC after RT were included. Specimens of normal urothelium and urothelium carcinoma were obtained from patients after radical cystectomy and nephro-ureterectomy. As shown in Table 4, no significant difference of basic characteristics was found in age (P = 0.63), gender (P = 0.42), tumor size (P = 0.51), histologic grade (P = 0.66), clinical stage (P = 0.24) and recurrence (P = 0.57) between primary UC group and UC after RT group.

**Table 4.** The characteristics of patients with primary UC and UC after RT

	Primary UC (%)	UC after RT (%)		*p*
Patients No.	60		54	
Age, years, median (range)	47 (38–69)		46 (34–65)	0.63
Gender				
Male	7		5	
Female	53		49	0.42
Tumor size				
<3 cm	36		32	
≥3 cm	24		22	0.51
Histologic grade				
I	26		24	
II	21		18	
III	13		12	0.66
Clinical stage				
T_a_-T_1_	38		36	
T_2_-T_4_	22		18	0.24
Recurrence				
-	44		14	
+	16		5	0.57

### Correlation between PPARγ expression and clinicopathologic features of patients with UC after RT

**Figure 5.**
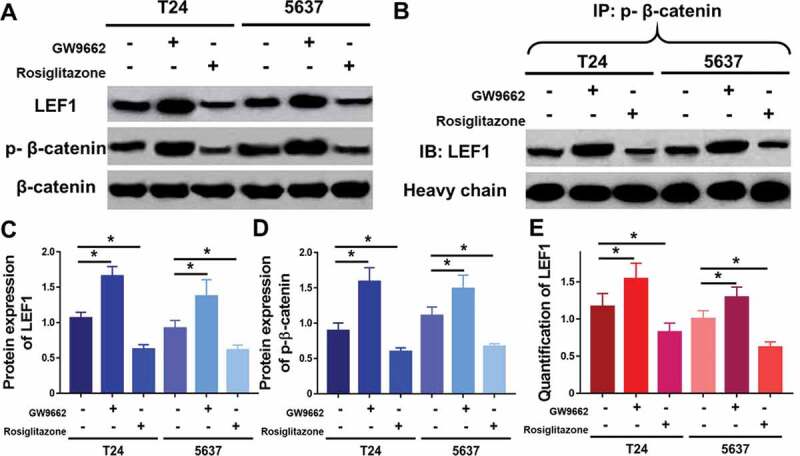
Expression of LEF1, phospho-β-catenin and total β-catenin were studied by western blot (a), and quantification of LEF1 expression (c) and phospho-β-catenin expression (d). Co-immunoprecipitation of LEF1 and phospho-β-catenin (b) and quantification of expression of LEF1 pulled by phospho-β-catenin (e). Two UC cell lines, T24 and 5637, were treated with PPARγ agonist Rosiglitazone (20 μM) or PPARγ antagonist GW9662 (20 μM). Experiments were repeated for three times. *p < 0.05

In order to validate the results of microarray, the expression levels of PPARγ were examined in normal urothelium and specimens of recipients with UC after RT, using immunohistochemistry staining and RT-PCR. As shown in Figure 5(a), all three samples from patients suggested a localization of PPARγ in the nucleus and lower expression of PPARγ in UC after RT group compared with normal urothelium group. The result in Figure 5(b) further confirmed the down regulation of PPARγ in UC after RT.

**Figure 6.**
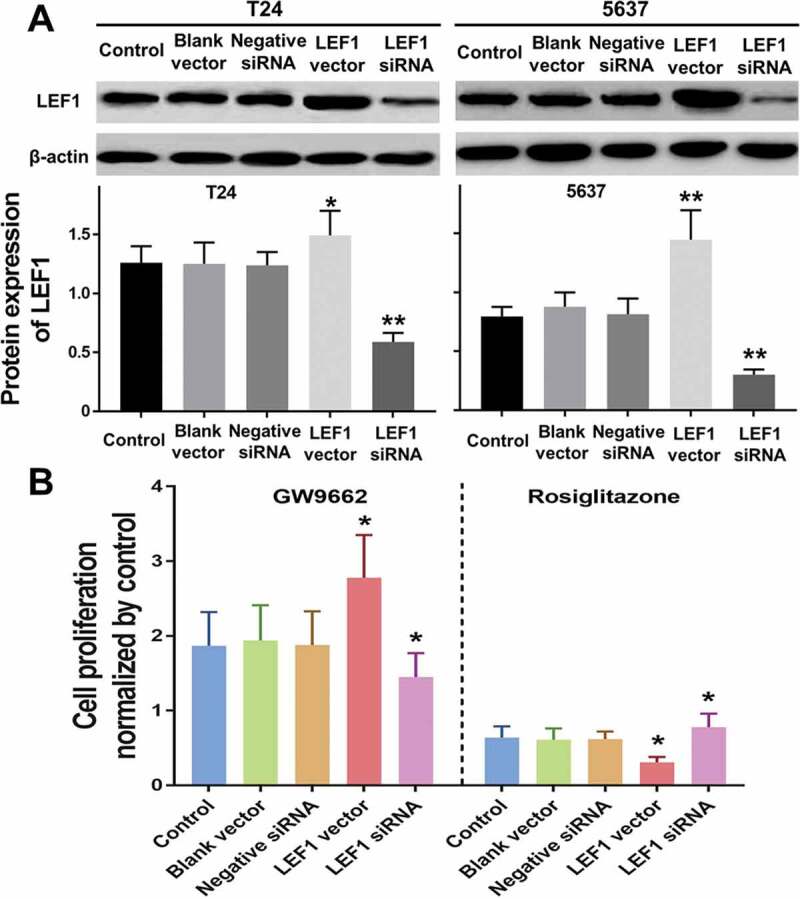
Expression of LEF1 in T24 and 5637 treated with PBS, blank vector, negative siRNA, expression vector containing the full-length cDNA for LEF1 and siRNA targeting LEF1 by western blot(a). Cell proliferation of T24 treated with following five agents respectively, and PPARγ agonist Rosiglitazone (20 μM) or PPARγ antagonist GW9662 (20 μM) normalized by control (b). Experiments were repeated for three times. *p < 0.05

Moreover, mRNA expression of PPARγ and RXRα were detected in patients with primary UC and UC after RT. As shown in Figure 6(a), the mRNA expression of PPARγ was significantly decreased in both primary UC (P < 0.05) and UC after RT (P < 0.01). Of note, the mRNA expression of PPARγ in UC after RT was much lower than that in primary UC (P < 0.05). Similarly, the mRNA expression of RXRα (Figure 6(b)) was significantly decreased in both primary UC and UC after RT (P < 0.01, both), but no significant differences were found in RXRα between two groups. These findings suggested that decreased expression of PPARγ might be closely related to the carcinogenesis of UC, especially in RT recipients.

In addition, we further investigated the correlation between PPARγ expression and clinicopathologic features of patients with UC after RT. By making comparisons of the PPARγ expression in protein and mRNA level, significant differences were found in tumor size, clinical stage, pathological grade, and recurrence, while age and gender showed no statistical difference (Table 5). Two side chi-square test was used for protein expression difference, and independent two-tail t-test was used for mRNA expression difference analysis. The following results indicated the PPARγ expression was possibly correlated with tumor size, clinical stage, pathological grade and recurrence, and may be involved in progression of patients with UC after RT.

**Table 5.** The correlation between PPARγ expression and clinicopathologic features of patients with UC after RT

	*n*	PPARγ protein expression		*p*	PPARγ mRNA expression	*p*
		-	+			
UC/RT	54	35	19		3.83 ± 0.31	
Urothelium	54	7	47	0.001	6.73 ± 0.65	0.000
Gender						
Male	5	3	2		3.76 ± 0.27	
Female	49	32	17	0.232	3.84 ± 0.29	0.783
Age (years)						
<60	43	28	15		3.82 ± 0.31	
≥60	11	7	4	0.512	3.84 ± 0.28	0.672
Tumor size						
<3 cm	32	19	13		4.74 ± 0.37	
≥3 cm	22	16	6	0.002	2.48 ± 0.24	0.000
Histologic grade						
I	24	13	11		4.88 ± 0.42	
II	18	12	6		3.57 ± 0.31	
III	12	10	2	0.001	2.07 ± 0.19	0.000
Clinical stage						
T_a_-T_1_	36	21	15		4.15 ± 0.38	
T_2_-T_4_	18	14	4	0.002	3.18 ± 0.34	0.005
Recurrence						
-	31	17	14		5.12 ± 0.49	
+	23	18	5	0.001	2.08 ± 0.22	0.000

### PPARγ inactivates LEF1/β-catenin signaling in UC cells

**Figure 7.**
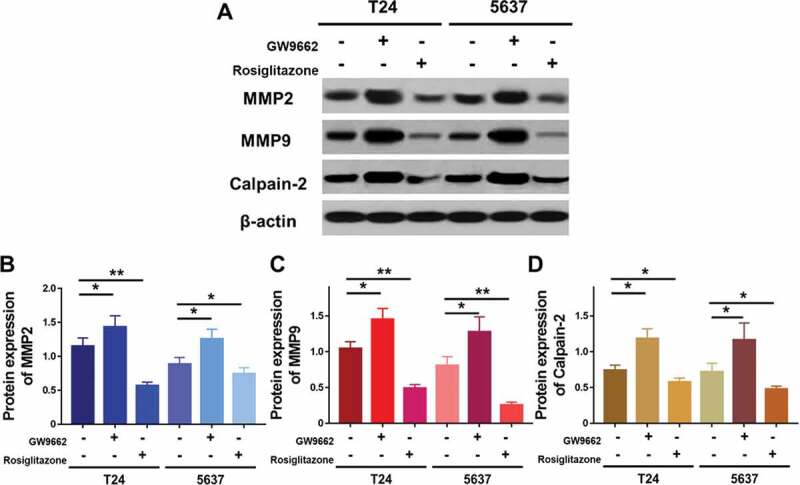
Expression of MMP2, MMP9 and calpain-2 in T24 and 5637 treated with PPARγ agonist Rosiglitazone (20 μM) or PPARγ antagonist GW9662 (20 μM) (a), and quantification of expression of MMP2 (b), MMP9 (c) and calpain-2 (d). Experiments were repeated for three times. *p < 0.05; **p < 0.01

To further study the molecular mechanisms of PPARγ in progression of UC, Rosiglitazone as PPARγ selective agonist and GW9662 as PPARγ antagonist were used to regulate the activity of PPARγ (Figure 7(a)). In both two UC cell lines including T24 and 5637, GW9662 (20 μM) could increase the expression of LEF1 and phospho-β-catenin (Figure 7(c,d)). In opposite, Rosiglitazone (20 μM) decreased the expression of LEF1 and phospho-β-catenin. Both drugs did not affect the expression of total β-catenin.

Furthermore, co-immunoprecipitation was performed to study whether an interaction between LEF1 and phospho-β-catenin existed. As shown in Figure 7(b,e), cells treated with GW9662 and pulled by phospho-β-catenin showed increased expression of LEF1 and cells treated with Rosiglitazone and pulled by phospho-β-catenin showed decreased expression of LEF1, indicating that LEF1 seemed to form a complex with phospho-β-catenin in both UC cells.

### PPARγ suppresses the proliferation of UC cells depending on LEF1 expression

**Figure 8.**
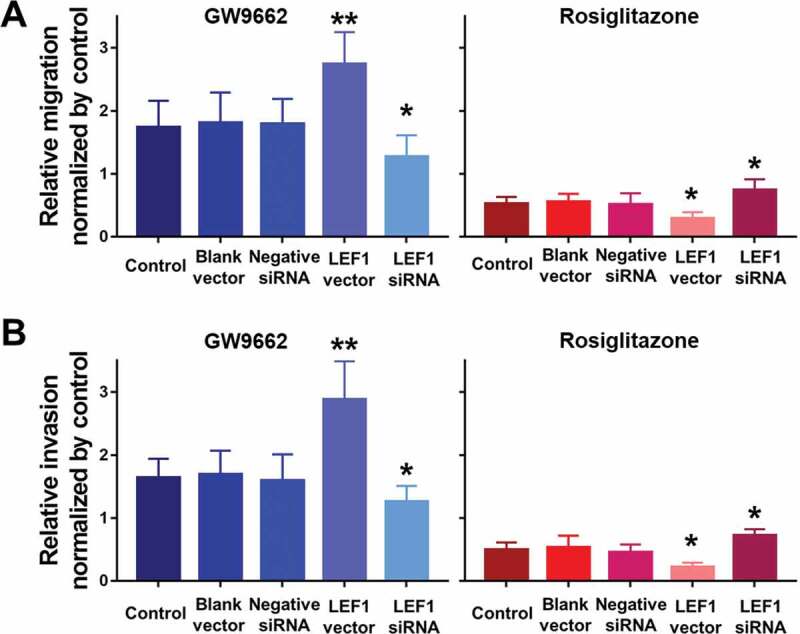
Migration (a) and invasion (b) of T24 treated with PBS, blank vector, negative siRNA, expression vector containing the full-length cDNA for LEF1 and siRNA targeting LEF1, followed by PPARγ agonist Rosiglitazone (20 μM) or PPARγ antagonist GW9662 (20 μM) normalized by control. Experiments were repeated for three times. *p < 0.05; **p < 0.01

Despite the PPARγ suppresses the proliferation and tumor growth of UC, the underlying mechanism remains unclear. To figure out the effect of LEF1 expression on PPARγ mediated anti-proliferation in UC, overexpression by transfection of LEF1 vectors and decreased expression by LEF1 siRNA were carried out. The successful transfections were confirmed as shown in Figure 8(a).

Cell proliferation of both two UC cells including T24 and 5637 were evaluated after treatment (only results of T24 cell are shown in Figure 8(b)). Regardless of the expression of LEF1, GW9662 as PPARγ antagonist promoted the proliferation of UC cells, while Rosiglitazone as PPARγ selective agonist suppressed the proliferation of UC cells, even when LEF1 was highly expressed, the cell proliferation of UC cells treated with GW9662 was much higher and treated with Rosiglitazone was much lower. In contrast, the trend was opposite in UC cells treated with LEF1 siRNA. These results indicated more effective PPARγ mediated regulation of cell proliferation.

### PPARγ suppresses the migration and invasion of UC cells depending on LEF1 expression

**Figure 9.**
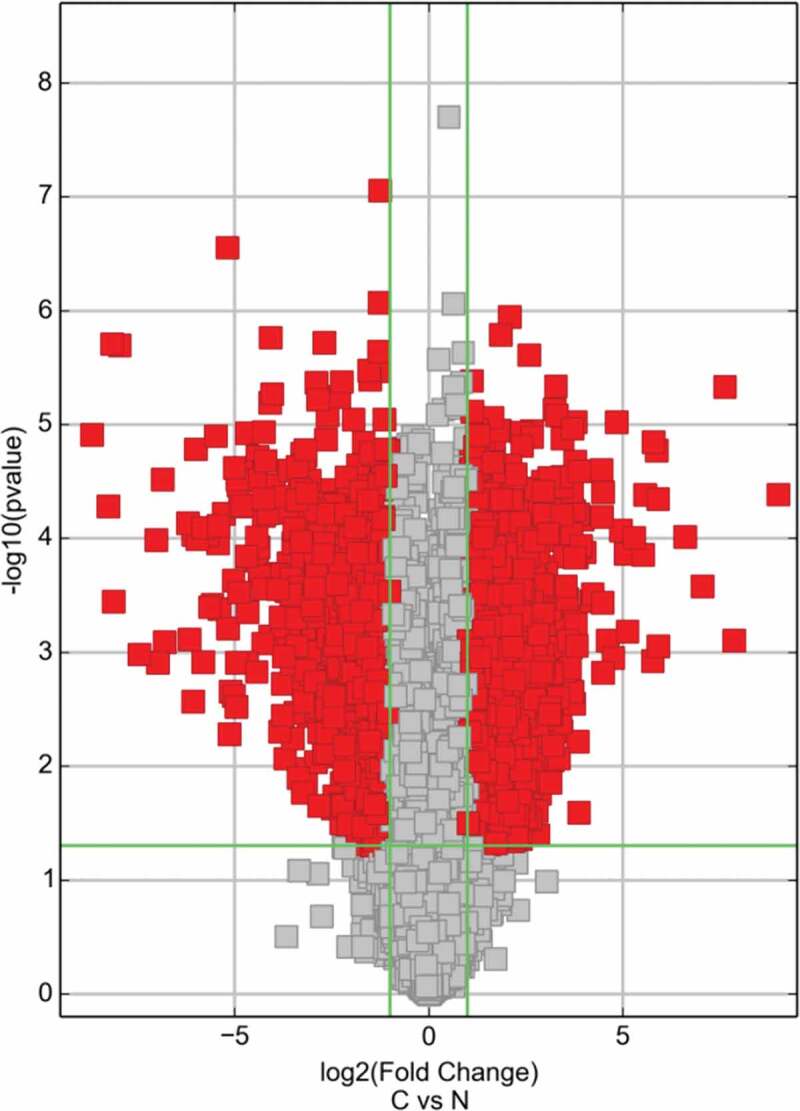
The mRNA expression of RT recipients in UC and its corresponding normal urothelial was screened by volcano plot filtering

**Figure 10.**
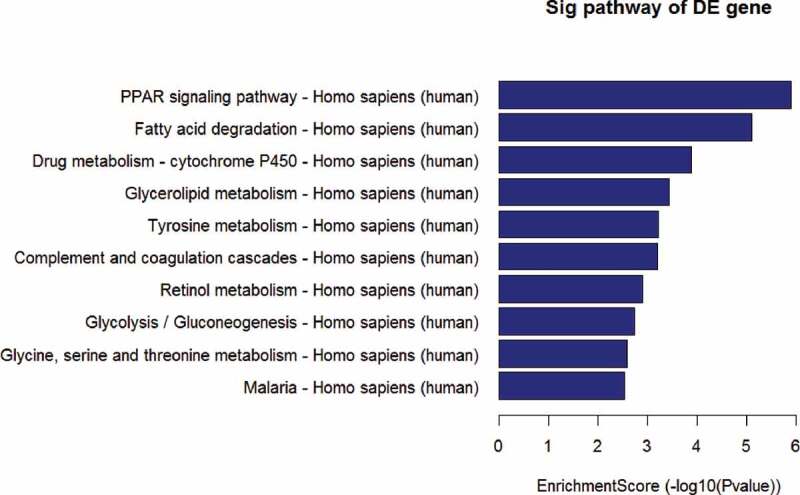
The enrichmentscore (-log10(Pvalue)) analyses of sig pathway of DE gene in different physiological process

To further study the effect of LEF1 expression on migration and invasion of UC cells, related protein expression was evaluated, and cell migration and invasion assay was used. As shown in Figure 9(a), GW9662 as PPARγ antagonist increased the expression of MMP2, MMP9, and calpain-2 in both UC cells. In contrast, Rosiglitazone as PPARγ selective agonist decreased the protein expression of MMP2, and calpain-2 (Figure 9(b,d)). Also, increased migration and invasion by GW9662 and decreased migration and invasion by Rosiglitazone of control, blank vector and negative siRNA as groups without any change of LEF1 expression were observed in T24 and 5637 cells (only results of T24 cell shown in Figure 10(a,b)), consistent with following results. Therefore, PPARγ could possibly have a big impact in the migration and invasion of UC after RT. In addition, UC cells of high LEF1 expression exhibited a greatly up-regulated sensibility to GW9662 and Rosiglitazone when compared to UC cells of low LEF1 expression, indicating that PPARγ suppressed the metastasis of UC cells depending on LEF1 expression.

## Discussion

In current study, the low expression of PPARγ in UC tissues, especially tumors of UC after RT may play a critical role and be closely related to the of UC after RT. PPARγ expression was negatively correlated with tumor size, clinical stage, pathological grade, and tumor recurrence, which could be used as a candidate biomarker for UC detection after RT.

A strong DNA binding domain exists in C-terminal of LEF1 and a transcription activator binding domain at its N-terminal, such as β-catenin [10]. Our previous results demonstrated that LEF1 expression increased significantly, which may be involved in the progression of RCC, so it might be used as a therapeutic target in advanced RCC [11]. However, the role of LEF1 in human UC needs further study. In this study, we found that LEF1 was a target of PPARγ, which could inactivate the LEF1/phosphor-β-catenin signaling, and LEF1/phosphor-β-catenin may have a big impact in the progression of UC after RT mediated by PPARγ after RT.

LEF1 has been reported to enhance the invasive and proliferative of melanoma cells [12]. In addition, LEF1 was found to mediate WNT/TCF Signaling on lung adenocarcinoma metastasis [13]. Consistently, we indicated that PPARγ inhibited the proliferation and metastasis of UC cells through the expression of LEF1/phosphor-β-catenin, especially in cells with over-expression of LEF1 (Figure 1). The migration and invasion of UC cells is considered to be an important multi-step process in tumor development. Bi *et al*. found that fascial proteins play a role in the migration and invasion of bladder urothelial carcinoma [14]. Our previous study found that the expression of MMP-3, 10, 12 and 13 was importantly up-regulated in tumors of UC after RT, which suggested strong tumor metastasis characteristics in UC cells after RT [1]. Considering the potential role of MMPs in the carcinogenesis and progression of UC after RT, we evaluated the expression of MMP2, MMP9 and calpain-2. Interestingly, decreased PPARγ significantly up-regulated the expression of MMP2, MMP9 and calpain-2, indicating that they are related to the migration and invasion of UC.

The inhibition of Wnt/beta-catenin signaling and up-regulation of PPARγ have been reported in ARVC. Gamma-catenin presents structural similarities with betacatenin. In transgenic mice, gamma-catenin translocates to the nucleus, competes with β-catenin, and inhibits the canonical Wnt/β-catenin signaling through the TCF/LEF transcription factors. This results in enhancing adipogenesis, thus summarizing the phenotype of the human ARVC [15].

Based on our previous studies, we found that patients with UC after RT exhibited higher ability of tumor proliferation, and invasion than patients with primary UC. Due to the follow-up care and strict routine checkups of RT recipients, most UC after RT could be detected more earlier. Radiotherapy induced cancer remains frequent, and the reasons for higher occurrence of UC after RT may be complex [16]. The application of immunosuppressants may lead to DNA damage may be related to the normal DNA repair mechanism [16]. Additionally, viral infections are linked to a number of cancers [16]. More importantly, the immune surveillance function to prevent the growth and development of malignant tumors could be impaired. In China, it has also been reported that the incidence rate of UC after RT was related to herbs containing aristolochic acid (AA) [17]. AA or its metabolites may be activated and to DNA resulting in DNA instability. The formation of AA-DNA adducts depends on the reductive activation of quinone oxidoreductase and cytochromes P450 [18]. Since the recipients of AA are more likely to develop UC, the relationship between PPARγ and AA needs to be investigated in future.

## Conclusion

In summary, our results demonstrated that PPARγ inhibits the proliferation and metastasis of this malignant tumor by inhibiting LEF1/β-catenin signaling transduction by RT and PPARγ. PPARγ may be a potential biomarker for the diagnosis of UC after RT. And the therapeutic strategy of restoring the expression of PPARγ may be a novel and promising method for the UC after RT.

## Highlights

1. PPARγ expression is significantly decreased in urothelial carcinoma (UC) after renal transplants (RT).
2. PPARγ expression is correlated with tumor size, clinical stage, pathological grade and recurrence of UC.
3. PPARγ inhibits the protein expression of MMP2, and calpain-2.
4. LEF1/β-catenin signaling is suppressed by PPARγ in UC cells, which mediates the inhibition of the proliferation and metastasis of UC cells.
